# Resveratrol Modifies Lipid Composition of Two Cancer Cell Lines

**DOI:** 10.1155/2020/5393041

**Published:** 2020-02-19

**Authors:** Luciana Gomes, Leidiane Viana, Jerson L. Silva, Claudia Mermelstein, Georgia Atella, Eliane Fialho

**Affiliations:** ^1^Laboratório de Alimentos Funcionais, Instituto de Nutrição Josué de Castro, Universidade Federal do Rio de Janeiro, Rio de Janeiro, Brazil; ^2^Instituto de Bioquímica Médica, Universidade Federal do Rio de Janeiro, Rio de Janeiro, Brazil; ^3^Instituto de Ciências Biomédicas, Universidade Federal do Rio de Janeiro, Rio de Janeiro, Brazil; ^4^Laboratório de Bioquímica de Lipídeos e Lipoproteínas, IBqM, Universidade Federal do Rio de Janeiro, Rio de Janeiro, Brazil

## Abstract

Resveratrol (Resv) offers health benefits in cancer and has been reported to modulate important enzymes of lipid metabolism. Studies of its effects on lipid composition in different subtypes of breast-cancer cells are scarce. Thus, we investigated the alterations in phospholipids (PL), fatty acids (FA), and lipid metabolism enzymes in two breast-cancer cell lines after Resv treatment. MCF-7 and MDA-MB-231 cells were treated with 80 and 200 *μ*M of Resv, respectively, for 24 hours. We analyzed PL with radiolabeled inorganic phosphate (^32^Pi) by thin-layer chromatography, FA by gas chromatography-mass spectrometry, and lipid metabolism enzymes (DGAT2, FAS, *ρ*ACC*β*, pAMPK*α*, and AMPK) by Western blot. Resv treated MDA-MB-231 phospholipids showed a reduction in phosphatidylcholine (63%) and phosphatidylethanolamine (35%). We observed an increase in eicosapentaenoic acid (EPA) (73%) and docosahexaenoic acid (DHA) (65%) in MCF-7 cells after Resv treatment. Interestingly, the same treatment caused 50% and 90% increases in EPA and DHA, respectively, in MDA-MB-231 cells. In MCF-7 cells, Resv increased the expression of *ρ*ACC*β* (3.3-fold) and AMPK*α*/*ρ*AMPK*α* (1.5-fold) and in MDA-MB-231 cells it inhibited the expression of *ρ*ACC*β* (111.8-fold) and AMPK*α*/*ρ*AMPK*α* (1.2 fold). Our results show that Resv modified PL and saturated and unsaturated FA especially in MDA-MB-231 cells, and open new perspectives to the understanding of the reported anticancer effect of Resv on these cells.

## 1. Introduction

Tumors share a common phenotype of uncontrolled cell proliferation and for this they must efficiently generate energy and biomass components to expand and disseminate. Cancer cells with high proliferation rates need lipids in larger quantities. It has been shown that cancer cells have increased uptake of lipids and lipoproteins or overactivation of endogenous lipid synthesis [1]. Cancer preclinical models and clinical trials have revealed the crucial role of lipid classes and molecular species in supporting tumor growth and metastatic dissemination [2].

Changes in lipid metabolic pathways to perturb lipid homeostasis through the targeting of enzymes, receptors, or bioactive lipids induce tumor regression and inhibit metastatic spread [1]. Drugs used in chemotherapy may target lipids. Among them, we can highlight tamoxifen, an antiestrogen used in first-line endocrine therapies for estrogen-positive (ER^+^) breast cancer, acting to inhibit phospholipid (PL) metabolism, leading to apoptosis [3]. Plant-derived bioactive compounds such as resveratrol (Resv), may also modulate lipid metabolism [4].

Resv (3,5,4′-trihydroxy-trans-stilbene) is the most extensively studied stilbene. It is found in grape skin, berries, peanuts, and some medicinal plants, where it draws attention due to its diverse biological and pharmacological actions, including antioxidation, anti-inflammation, antidiabetic potencies, and anticarcinogenic characteristics. Usually, the effect of resveratrol in breast-cancer cells is attributed to cell death via multiple pathways including apoptosis, cell-cycle arrest in the S phase, and autophagy [5, 6]. Several studies have focused on its role in lipid composition in relation to cardioprotection, obesity, and diabetes situations [7–9], but in breast-cancer cells, studies are rare [10, 11].

More studies are needed to improve the therapeutic interventions for the treatment of human breast cancers [12–14]. Different subtypes of breast-cancer cells vary with respect to prognosis, aggressiveness, and response to therapies. MCF-7 and MDA-MB-231 cell lines are widely used in biomedical studies since they provide two distinct phenotypical characteristics. The MDA-MB-231 cell line, a triple-negative breast-cancer cell which does not express estrogen receptor (ER), progesterone receptor (PR), or human epithelial receptor 2 (HER2), is considered to be representative of a highly aggressive type of cancer cell, whereas MCF-7 expresses ER and PR and is considered a less aggressive cancer cell.

The differences between these two breast-cancer cell lines are reflected not only in their response to chemotherapeutics but also in their response to Resv. It has been reported that Resv can differentially regulate cell death in these two cell lines [5, 15]. Although the importance of lipid metabolism for the maintenance of tumor cells and the modulation of fat homeostasis by resveratrol [1, 4] are known, interestingly, this relationship has not yet been studied in these two cell lines. Thus, the present study aims to analyze the effect of Resv on phospholipids, fatty acids, and lipid metabolism enzymes in MCF-7 and MDA-MB-231 cells. Our hypothesis is that Resv will affect MCF-7 and MDA-MB-231 human breast-cancer cell lines differently due to their distinct phenotypical and biochemical characteristics, such as the expression of ER and PR receptors and their migratory abilities.

## 2. Materials and Methods

### 2.1. Cell Cultures

MCF-7 and MDA-MB-231 cell lines were obtained from ATCC (American Type Culture Collection). Both cells were grown in DMEM (Dulbecco's Modified Eagle's medium) supplemented with 10% fetal bovine serum and 1% penicillin and streptomycin solution. The cells were maintained at 37°C in a humidified atmosphere of 5% CO_2_. Cultures with 70–80% confluence were treated with *trans*-resveratrol (Sigma-Aldrich, USA). DMSO (dimethyl sulfoxide, 0.1%) was used to dissolve Resv and therefore was used as a control. Previous work from our group [5] showed that these two cell lines presented different sensitivities to resveratrol in terms of cell viability, and thus we decided to use in the present work 80 *μ*M resveratrol in experiments with MCF-7 cells and 200 *μ*M resveratrol in MDA-MB 231 cells.

### 2.2. Lipid Extraction

MCF-7 and MDA-MB-231 cell cultures with 70–80% confluence were treated with resveratrol or DMSO (control) for 24 h; supernatants were discarded, and the total number of cells was counted using a Neubauer cell chamber. Then, 1.0 × 10^7^ cells were subjected to lipid extraction as described previously by Bligh and Dyer [16]: methanol : chloroform : water (2 : 1 : 0.8 v/v) solution was added to the cells. Cells were vortexed briefly every 5 min during 1 h and centrifuged at 4°C for 20 min at 3000 rpm. The lipid-containing supernatants were separated from the pellets. This process was performed twice, and the supernatants were combined. The supernatants were added to water : chloroform (1 : 1) and vortexed for 30 s. The samples were centrifuged (3000 rpm/30 min), the lipid phase was separated, and the solvent was evaporated by an N_2_ stream. Lipid extracts were resuspended in chloroform for analysis by gas chromatography-mass spectrometry (GC-MS).

### 2.3. Phospholipid Content Analysis with Radiolabeled Inorganic Phosphate (^32^Pi)

MCF-7 and MDA-MB-231 (2 × 10^5^) cells were cultivated in 24-well plates, incubated with Resv and DMSO 0.1% (control) in serum-free DMEM medium supplemented with ^32^Pi (1.5 mCi/mL) for 0, 2, 8, 24 h. Then cells were subjected to lipid extraction as described above and the associated radioactivity was measured by liquid scintillation counting [17]. We have recently shown that concentrations of resveratrol above 50 *μ*M induce a significant decrease (∼40–50%) in cell viability in MCF-7 cells, while the same concentration of resveratrol causes only a minor decrease (∼10–40%) in cell viability in MDA-MB-231 cells [18]. To normalize the effects of resveratrol in cell viability, we used 80 *μ*M of resveratrol in MCF-7 cells and 200 *μ*M of resveratrol in MDA-MB-231 cells.

### 2.4. Phospholipid Classes Separated by Thin-Layer Chromatography (TLC)

Phospholipid extracts were analyzed by one-dimensional TLC on silica-gel plates (Silica Gel 60F254, Merck, Frankfurt, DS, Germany). After application of the samples to the plate, the run was performed with chloroform : acetone : methanol : acetic acid : distilled water (40 : 15 : 13 : 12 : 8) [19]. Radiolabeled lipids were visualized by exposing them to a Kodak X-OMAT film (Eastman Kodak Comp., Rochester, NY, USA) for approximately 2 weeks at −70°C, using a TranScreen phosphor (Eastman Kodak Comp., Rochester, NY, USA). The spots were cut out, reextracted, and the radioactivity associated with each spot was analyzed by liquid scintillation.

### 2.5. Fatty-Acid Profile by GC-MS Analysis

Sterols and fatty acids were extracted as described above. For analysis of sterols, aliquots of the total lipid extracts dried in an N_2_ stream were resuspended in 25% alcoholic potash, vortexed, and heated at 85°C in a water bath for 1 h. Then, water (1 mL) and heptane (2 mL) were added, vortexed, centrifuged, and dried under an N_2_ stream. After, *N*,O-Bis(trimethylsilyl)trifluoroacetamide : trimethylchlorosilane (99 : 1) and pyridine were added. The samples were heated at 65°C in a water bath [20]; then 1 *μ*L was used for analysis. For fatty-acid studies, the total dry lipid extract was resuspended in 1 mL toluene and incubated overnight with 1% sulfuric acid in methanol at 50°C. After that, 1 mL 5% sodium chloride was added and the sample was briefly dried under an N_2_ stream, dissolved in toluene and 1 *μ*L was used for analysis. The lipids were separated by GC-MS using a gas chromatograph (Shimadzu, GP2010 Plus) equipped with an HP Ultra 2 column (Agilent, 25 m × 0.20 mm × 0.33 *μ*m) coupled to a mass spectrometer (Polaris Q, Thermo Fisher Scientific). The injector was maintained at 250°C and the temperature of the column was increased from 40°C to 160°C, with a heating rate of 30°C/min, from 160°C to 233°C, with a heating rate of 1°C/min, from 233°C to 300°C, with a heating rate of 30°C/min and maintained at 300°C for 10 min. Helium was used as the carrier gas, with a linear velocity of 32.9 cm/sec. One microliter of the sample was injected in the chromatograph. The molecules were ionized by electron impact (EI) at 70 eV at 240°C. The spectra were collected in the 30–400 *m*/*z* range, and a chromatogram was generated by plotting the detected spectra of fragmented-ion species for *m*/*z* 41, 43, and 55. A calibration curve was recorded using FAME 37 methylated FA mix standard (Supelco, Sigma-Aldrich) and sterols and fatty acids were identified by comparison.

### 2.6. Preparation of Protein Extracts

For Western blots, cells were washed with PBS and lysed in liquid nitrogen. Cells were then scraped using lysis buffer (5 mM Tris-HCl, 10 mM ethylenediamine tetraacetic acid, 5 mM sodium fluoride, 1 mM sodium orthovanadate, 1 mM phenylarsine oxide, 1 *μ*M okadaic acid and 1 mM phenylmethylsulfonyl fluoride; pH 7.4) with a cocktail of protease inhibitors (Sigma). The lysate was collected, sonicated, and cleared by centrifugation at 8000 rpm for 5 min at 4°C. The supernatant (total cell lysate) was then collected, aliquoted, and stored at −80°C. The protein concentration was determined according to Lowry et al. [21]. Bovine serum albumin was used as a standard.

### 2.7. Lipid Metabolism Enzymes Analysis by Western Blot Assay

Equal amounts of total cellular proteins (60 *μ*g) were applied on sodium dodecyl sulfate-polyacrylamide gel electrophoresis (SDS-PAGE) and transferred onto polyvinylidene difluoride (PVDF) membranes (Immobilon P, Millipore, Bedford, MA). Membranes were blocked for 2 h in Tris-buffered saline containing 1% Tween 20 (TBS-T) and 5% nonfat milk and incubated for 2 h with the primary antibody (1 : 1000). The membranes were then washed with TBS-T and incubated with a peroxidase-conjugated secondary antibody (1 : 1000) for 2 h. The antibodies used were as follows: anti-*ρ*AMPK*α* (#2535), from Cell Signaling Technology and anti-DGAT2 (sc-32399), anti-fatty-acid synthase (FAS, sc-20140), anti-*ρ*ACC*β* (sc-30446), anti-AMPK*α*1 (sc-398861), and anti-GAPDH (sc-32233), from Santa Cruz Biotechnology. Protein bands were visualized with the enhanced chemiluminescence (ECL) kit (Amersham, U.K.) using C-DiGit Chemiluminescent Western Blot Scanner (LI-COR Biotechnology, USA). Images were analyzed using IMAGE J 1.50i software (NIH, USA). The results were expressed as arbitrary units, calculated by the fraction of pixels presented by each band relative to GAPDH or AMPK*α*1.

### 2.8. Statistical Analysis

Quantitative data represent the mean values with the respective standard error of the mean (SE) corresponding to three or more replicates from independent experiments. Data were analyzed by one-way analysis of variance (ANOVA) using the post hoc multiple comparisons Tukey test. GraphPad Prism software (GraphPad Software, Inc, La Jolla, CA, USA), version 6.01, was used for statistical analyses. Differences were considered statistically significant when *p* < 0.05.

## 3. Results

### 3.1. Resveratrol Alters the Total Phospholipid (PL) Content in Breast-Cancer Cells

**Figure 1:**
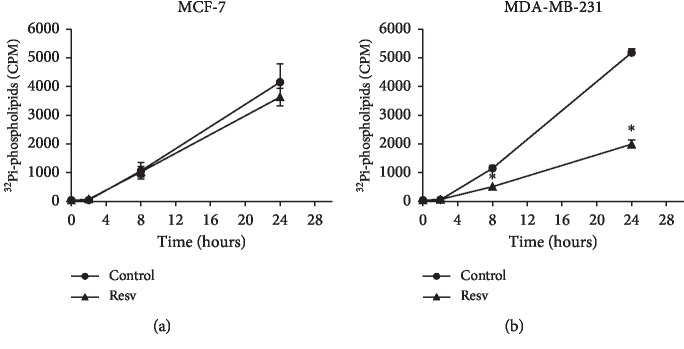
Resveratrol effect on the total phospholipid content in MCF-7 and MDA-MB-231 cells. Results are expressed as counts per min (CPM) of synthesized ^32^Pi-phospholipids at the indicated times. Error bars represent mean ± SD, *n* = 2-3. ^*∗*^Statistically different (*p* < 0.05).

To verify the effect of Resv on PL content, ^32^Pi was used. MCF-7 cells exhibited a slight increase in total PL content (Figure 1) with 2 h (77 CPM) of Resv treatment compared to control (51 CPM). After 8 and 24 h, PL biosynthesis was similar to control, and the values of 1035 CPM (8 h) and 3637 CPM (24 h) were found for treated MCF-7 cells and 1067 CPM (8 h) and 4153 CPM (24 h) for control cells. On the other hand, Resv modified PL biosynthesis in MDA-MB-231 cells. After 8 h, we observed a reduction of PL content (518 CPM) in relation to control (1155 CPM), and this difference in biosynthesis was accentuated after 24 h of treatment (1992 CPM for treated and 5181 CPM for control cells).

**Figure 2:**
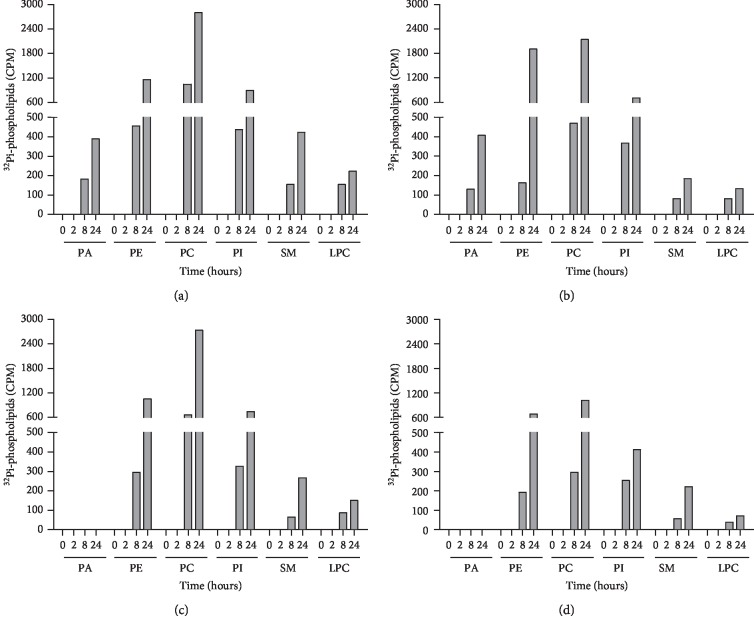
Resveratrol effect on phospholipid biosynthesis in MCF-7 and MDA-MB-231 cells. MCF-7 cells ((a) control, (b) treated) and MDA-MB-231 cells ((c) control, (d) treated). Results are expressed as counts per min (CPM) of synthesized ^32^Pi-phospholipids at the indicated times. PA: phosphatidic acid; PE: phosphatidylethanolamine; PC: phosphatidylcholine; PI: phosphatidylinositol; SM: sphingomyelin; LPC: lysophosphatidylcholine. *n* = 1.

**Figure 3:**
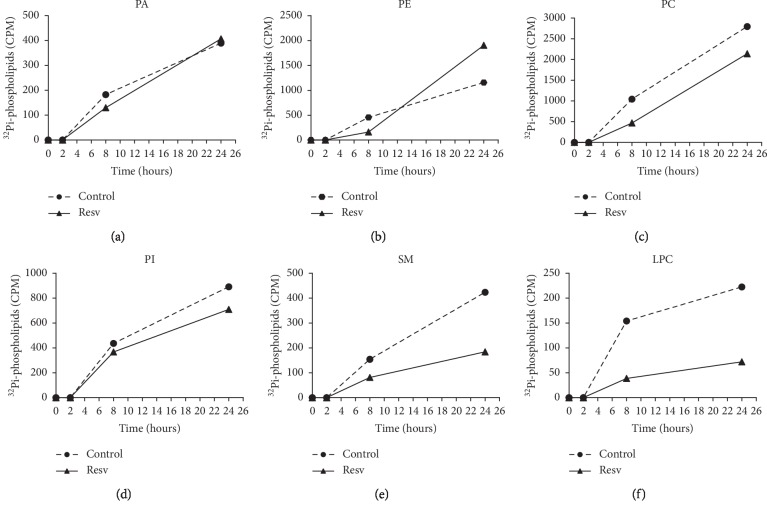
Time course of phospholipid biosynthesis in MCF-7 cells treated with resveratrol. Results are expressed as counts per min (CPM) of ^32^Pi-phospholipids synthesized over 24 h. PA: phosphatidic acid; PE: phosphatidylethanolamine; PC: phosphatidylcholine; PI: phosphatidylinositol; SM: sphingomyelin; LPC: lysophosphatidylcholine. *n* = 1.

In addition, the PL classes were verified by TLC after 24 h. In controls of MCF-7 (Figure 2(a)) and MDA-MB-231 (Figure 2(c)) cells, PE, PC and PI were synthesized to higher concentrations, compared to SM and LPC. Only MCF-7 cells presented PA. Resv treatment of MCF-7 cells (Figures 2(b) and 3) reduced PC (23%-2141 CPM for treated and 2796 CPM for control cells) and PI (20%-710 CPM for treated and 892 CPM for control cells) biosynthesis. SM and LPC biosynthesis presented a pronounced decrease of 57% (184 CPM for treated and 424 CPM for control cells) and 40% (133 CPM for treated and 223 CPM for control cells) respectively, while PE showed an increase of 65% after 24 h (1905 CPM for treated and 1156 for control cells).

**Figure 4:**
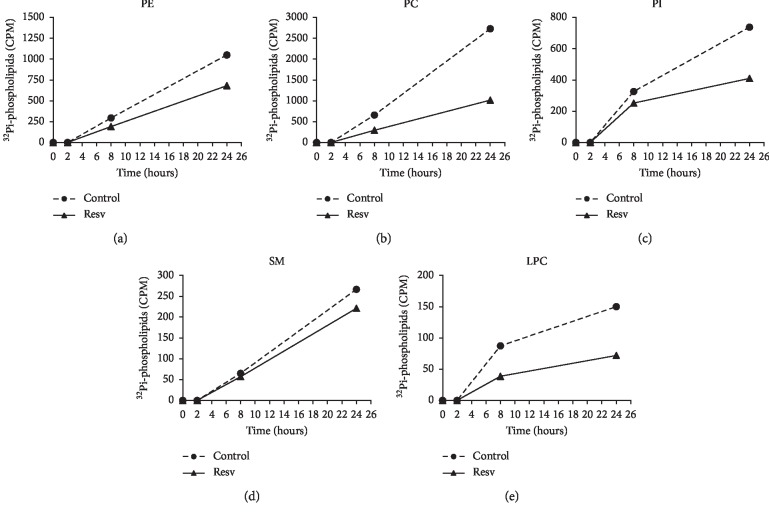
Time course of phospholipid biosynthesis in MDA-MB-231 cells treated with resveratrol. Results are expressed as counts per min (CPM) of ^32^Pi-phospholipids synthesized over 24 h. PA: phosphatidic acid; PE: phosphatidylethanolamine; PC: phosphatidylcholine; PI: phosphatidylinositol; SM: sphingomyelin; LPC: lysophosphatidylcholine. *n* = 1.

MDA-MB-231 cells showed a decrease in PL class biosynthesis in 8 h, accentuated at 24 h (Figures 2(d) and 4). PC exhibited the most remarkable reduction of 63% (1020 CPM for treated and 2730 for control cells) followed by LPC with 52% (72 CPM for treated and 150 for control cells), PI with 44% (411 CPM for treated and 739 for control cells), and PE with 35% (682 CPM for treated and 1051 for control cells). At 0 and 2 h no incorporation of ^32^Pi was observed in either of the cell lines, suggesting that at these time intervals and under these conditions there was no synthesis of PL. Interestingly, when Resv treated MDA-MB-231 cells were compared to Resv treated MCF-7 cells, we observed a marked decrease in PE (2.8-fold) and PC (2.0-fold) biosynthesis at 24 h.

### 3.2. Fatty-Acid Profile Modified in MCF-7 and MDA-MB-231 by Resveratrol Treatment

Saturated and unsaturated FA contents were analyzed by Gas Chromatography-Mass Spectrometry (GC-MS). A total of 13 saturated FAs was identified (Table 1). In MCF-7 cells before exposure to Resv, palmitic acid (C16 : 0) was present in greater amount followed by stearic acid (C18 : 0), lauric acid (C12 : 0), and myristic acid (C14 : 0) with concentrations of 0.745, 0.583, 0.212, and 0.158 *μ*g/*μ*L, respectively. Treatment with Resv induced a reduction in the content of tridecanoic acid (C13 : 0) by 52%, while other saturated FA contents increased, especially stearic acid (C18 : 0, 45%) and lauric acid (C12 : 0, 43%). Heneicosanoic acid (C21 : 0) and tricosanoic acid (C23 : 0) were found only in MCF-7 control cells and in minor amounts.

MDA-MB-231 untreated cells also exhibited greater amounts of stearic acid (C18 : 0) and palmitic acid (C16 : 0) (0.997 ± 0.587 and 0.814 ± 0.235 *μ*g/*μ*L, respectively) and these values were higher than in MCF-7 cells (0.583 ± 0.285 and 0.745 ± 0.100, respectively). Comparison between the median of the relative increase of stearic acid (C18 : 0) over all FAs showed that MDA-MB 231 cells did not change the amount of C18 : 0 (∼50% in both control and Resv), whereas MCF-7 cells displayed a minor decrease in the amount of C18 : 0 from ∼35% in control (DMSO) to ∼30% after Resv treatment. On the other hand, lignoceric acid (C24 : 0), hexacosanoic acid (C26 : 0), and docosanoic acid (C22 : 0) showed accentuated increase in their contents (87%, 53%, and 50%, respectively) after Resv treatment, although they were found in smaller amounts. C13 : 0, C21 : 0, and C23 : 0 were not found in MDA-MB231 cells, but they were present in untreated MCF-7 cells.

MCF-7 and MDA-MB-231 cells differed in total saturated FA content, with minor amounts in MCF-7 (1.831 *μ*g/*μ*L) and slightly more in MDA-MB-231 (2.032 *μ*g/*μ*L). However, resveratrol-treated cells showed similar contents of saturated FA, with 2.408 *μ*g/*μ*L in MDA-MB231 and 2.352 *μ*g/*μ*L in MCF-7 cells.

In this study 15 unsaturated FAs were identified (Table 2). The most abundant unsaturated FA in the MCF-7 cells was oleic acid (C18:1n9c), followed by elaidic acid (C18 : 1n9t) and palmitoleic acid (C16 : 1n7) at concentrations of 0.542, 0.419, and 0.223 *μ*g/*μ*L, respectively. Under Resv exposure these three unsaturated FAs maintained their predominance and the contents of oleic acid (C18 : 1n9c) and palmitoleic acid (C16 : 1n7) increased by 53% and 13%, respectively. However, elaidic acid (C18 : 1n9t) showed a 20% decrease with Resv. The other unsaturated FAs were found at lower levels, although some showed a marked increase in their content in the presence of Resv, particularly EPA (C20 : 5n3) with 73% and DHA (C22 : 6n3) with 65%.

Similar to MCF-7 cells, MDA-MB-231 cells also exhibited higher oleic acid (C18 : 1n9c) and elaidic acid (C18 : 1n9t) content, followed by arachidonic acid (C20 : 4n6) with levels of 0.460, 0.162, and 0.123 *μ*g/*μ*L, respectively. Resv treatment favored considerable changes in 8, 11, 14-eicosatrienoic acid (C20 : 3n6), C20 : 4n6, C22 : 6n3, and Cis-10-heptadecanoic acid (C17 : 1n7), increasing their concentrations by 141%, 91%, 90%, and 65%, respectively. Major differences in FA profile were found in MDA-MB-231 cells, which showed a 53% increase in unsaturated FA content, in contrast to the modest increase in saturated FA (16%). Thus, unsaturated FA content in MDA-MB-231 and MCF-7 was similar after Resv treatment, with values of 1.832 and 1.881 *μ*g/*μ*L, respectively.

### 3.3. Effect of Resveratrol on Expression of Lipid Metabolism Enzymes

**Figure 5:**
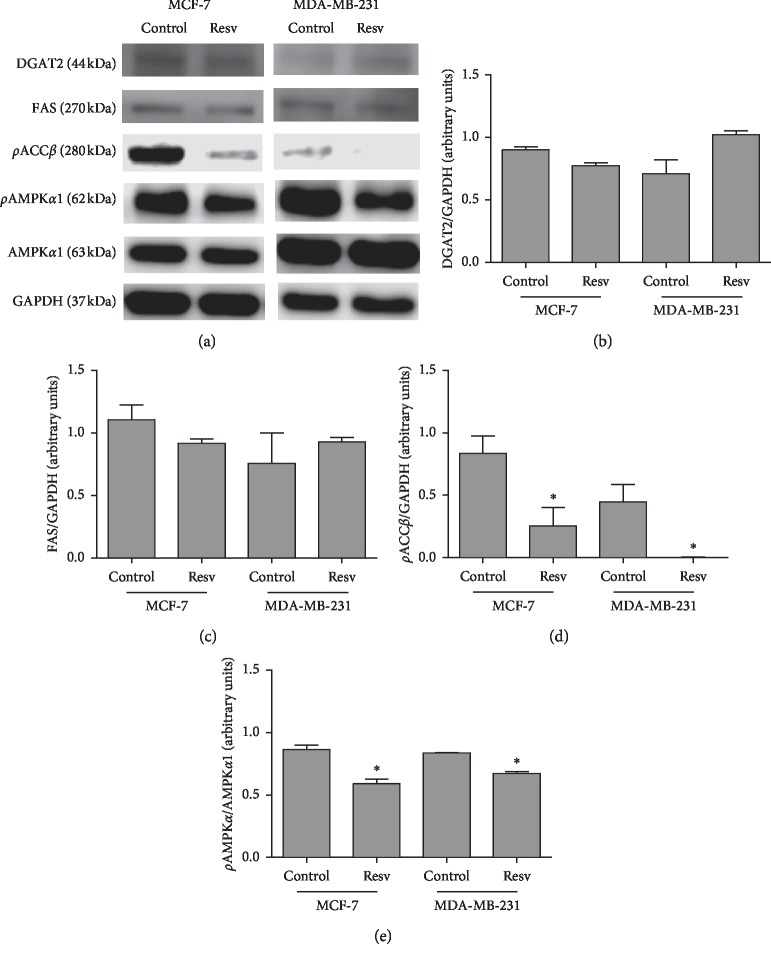
Resveratrol effect on the expression of lipid metabolism enzymes in MCF-7 and MDA-MB-231 cells. (a) Western blot analysis of DGAT2, FAS, *ρ*ACC*β*, pAMPK*α*, and AMPK*α*1. (b-e) Graphs represent the densitometric analysis, calculated by the fraction of pixels in each band relative to the pixels in GAPDH (loading control). Error bars represent mean ± S.E.M., *n* = 3. ^*∗*^ Statistically different (*p* < 0.05). (b) DGAT2—Acyl-CoA : diacylglycerol acyltransferase 2, (c) FAS—Fatty-acid synthase, (d) *ρ*ACC*β* - Phosphorylated acetyl-CoA carboxylase *β*, and (e) *ρ*AMPK*α*/AMPK*α*—phosphorylated AMP-activated protein kinase *α*/AMP-activated protein kinase *α*.

To evaluate a possible modulation in lipid metabolism enzymes by resveratrol, Western blot analysis was performed (Figure 5) and DGAT2, FAS, *ρ*ACC*β*, and AMPK*α*/*ρ*AMPK*α* were investigated after 24 h of treatment with Resv. MCF-7 cells treated with Resv showed slightly lower levels of DGAT2 and FAS expression compared to control cells, as well as significantly lower levels of *ρ*ACC*β* and AMPK*α*/*ρ*AMPK*α* (3.3- and 1.5-fold, respectively). Resv treatment also induced substantial inhibition of ACC*β* phosphorylation in MDA-MB-231 cells.

## 4. Discussion

Resveratrol (Resv) is a bioactive compound able to induce changes in membrane lipids [22]. Our results indicate that Resv greatly alters PL content in MDA-MB-231 cells, which in the absence of Resv exhibited less PL biosynthesis compared to MCF-7 cells. Classes of PL biosynthesis were markedly modified by Resv treatment in both cell types. In the last few years, several studies have emerged with the proposal to elucidate the differences between tumor and nontumor cells and to use PLs as biomarkers in the early diagnosis of diverse types and grades of tumors [23–26]. PL levels in nontumor cells can be 3-fold less than in cancer cells [27].

Although it is essential to characterize the differences between tumor cells and nontumor cells, studies that help to elucidate the effect of bioactive compounds such as Resv which have the potential to be used as an adjuvant in chemotherapy are necessary [5]. Most of the work published in this context studies the action of lipids added to cells grown in culture [28–31] and not those synthesized by the cells, after a nonlipid stimulus.

In addition to their role in cell survival, lipids are important signaling molecules. Sphingolipids such as SM are important components of *de novo* lipid synthesis, playing a key role in cancer development [32]. Phosphoinositides are second messengers that transmit information from HER to the cellular machinery, recruiting effector proteins to specific compartments of the cell membrane. The main representative of the phosphoinositide class is phosphatidylinositol (3,4,5)-trisphosphate, which is produced by PI3K in response to growth-factor signals and activates Akt. It is worth remembering that both HER and PI3K/Akt have been shown to be targets of Resv action [4, 33].

Some significant differences in lipid profile between nontumor cells and tumor cells have been described, such as the lower percentage of PE and the occurrence of PA exclusively in tumor cells [23]. These data differ from ours: we did not detect PA in MDA-MB231 cells. In addition, we observed that Resv increased the content of PE in MCF-7 cells. Dória et al. [23] found that the most pronounced differences between the three cell types (nonmalignant mammary epithelial cells MCF10A, nonmetastatic breast-cancer cells T-47D, and metastatic breast-cancer cells MDA-MB-231) in their study occurred in the FA composition profile of each PL. For example, the metastatic potential was associated with higher concentrations of PC (O-16 : 0/18 : 1), PC (O-16 : 0/20 : 1), and PI (22 : 5/18 : 0) in MDA-MB-231. In our study, we found that Resv treatment decreased biosynthesis of PLs such as PC and PE in MDA-MB-231 cells.

He et al. [25] found increased PC and reduced SM and PI contents in six breast-cancer cell lines (including MCF-7 and MDA-MB-231 cells), relative to the control, a nontumoral cell. The authors emphasize that the significant increase in monounsaturated lipids may be associated with the degree of malignancy of breast-cancer cells [24, 25]. Our results show that Resv treatment of MDA-MB-231 cells caused a decline in the ratio of saturated to unsaturated fatty acids toward values similar to those of MCF-7, a cell with less aggressive characteristics.

Analyzing the interaction of Resv with membrane glycerophospholipids, Olas and Holmsen [34] showed that the bioactive compound at 2.5 and 50 *μ*g/mL appears to be located between the molecules of the phospholipid monolayer, penetrating more easily between serines and ethanolamines. It is possible that one of the hydroxyls of Resv is attracted to the carboxyl group of the serine, explaining its greater affinity for PS than for PE and PC. However, such a condition is also influenced by the length of the acyl group, the degree of instability, and the nature of the hydrophobic head of the PL.

In rat hepatocytes, the effect of Resv on the reduction of FA synthesis is dose dependent. In these cells, 25 *μ*M Resv for 30 min decreases PE, PI, and PC. Among the enzymes that participate in lipid metabolism, the activity of acetyl-CoA carboxylase (ACC) was significantly affected by the phytochemical, and the same occurred for the FAs C14 : 0, C16 : 0, C18 : 0, and C18 : 1 [35]. Often, Resv is related to the inhibition of lipogenesis in nontumor cells, such as in adipocytes. Their molecular targets, in this case, may be ACC, Akt, and AMPK [36, 37]. However, our results showed that Resv did not inhibit lipid synthesis in the breast-cancer cells MCF-7 or MDA-MB-231.

When we analyzed by Western blotting the expression of five enzymes involved in lipid metabolism (DGAT2, FAS, *ρ*ACC*β*, *ρ*AMPK*α*, and AMPK*α*), we found that Resv reduced the phosphorylation of ACC in both cell types, with this action being more pronounced in MDA-MB-231 cells. Furthermore, the phytochemical negatively regulated AMPK*α* expression. Pandey et al. [38] investigated FAS under the effect of Resv in MCF-7 and MDA-MB-231 cells and found divergent results from ours. They found that cells treated with 50 and 100 *μ*M Resv showed a significant reduction in FAS and, consequently, in the total lipid content. Additionally, they found that the decrease in FAS caused the induction of DAPK2 and BNIP3 proapoptotic genes, significantly increasing the percentage of cells in apoptosis. Importantly, here we report that Resv induces an increase in all FAs concomitant with a slight decrease in the protein expression of FAS. Since FAS is an enzyme involved in lipid metabolism, we cannot exclude the hypothesis that the activity of FAS, and not its expression, may be increased after Resv treatment. Further experiments are needed to test the activity of FAS in breast-tumor cells after Resv exposure.

Our study reveals that the treatment with Resv regulates ACC*β* and AMPK components of the metabolism of lipids, favoring the increase of FA content. Although the increase in FA biosynthesis is an undesirable condition in tumor cells, closely related to their survival, proliferation, metastasis, and malignancy, other issues must be considered [1]. The FA C20 : 4n6, which in our study exhibited an increase in its concentration in MDA-MB-231 cells in the presence of Resv, is an inducer of migration and invasion in these same cells. However, this action occurs through the pathway that is dependent on PI3K/Akt, two molecules that are inhibited by the action of Resv [39]. Thus, it is necessary to investigate other molecules involved in lipid metabolism and their modulation by Resv to clarify the effects of Resv in the regulation of lipid profiles in MCF-7 and MDA-MB-231 cells.

A possible explanation for our results showing that resveratrol had a more significant effect on lipid composition in MDA-MB-231 cells than in MCF-7 cells could be related to the higher concentration of resveratrol used in our study in MDA-MB-231 cells (200 *μ*M) as compared to MCF-7 cells (80 *μ*M). As recently shown by our group, these different concentrations were used to normalize the effects of resveratrol in cell viability in the two cell lines [18].

## 5. Conclusions

In conclusion, our results demonstrate for the first time that resveratrol effects on lipid composition are more evident in MDA-MB-231 cells than in MCF-7 cells. In both cancer cells, Resv treatment is associated with increased contents of unsaturated FAs, including DHA and EPA. Curiously, FA content in MDA-MB-231 cells treated with Resv approached the total amount observed in treated MCF-7 cells. This study represents an initial step for future studies that could explore the relationship between modifications in lipid composition caused by Resv and the anticancer potential of this bioactive compound.

## Figures and Tables

**Table 1:** Saturated lipid profile of MCF-7 and MDA-MB-231 cells after resveratrol treatment.

FA ID	Lipid name	R.T.	MCF-7		MDA-MB-231	
			Control	RESV	Control	RESV
			(*μ*g/*μ*L)		(*μ*g/*μ*L)	
C12 : 0	Lauric acid	9.774	0.212^a^	0.304^a^	0.015^a^	0.013^a^
C13 : 0	Tridecanoic acid	11.740	0.023^a^	0.011^a^	ND	ND
C14 : 0	Myristic acid	14.676	0.158 ± 0.011	0.184 ± 0.096	0.081 ± 0	0.106 ± 0.014
C15 : 0	Pentadecanoic acid	18.323	0.035 ± 0.002	0.036 ± 0.020	0.013 ± 0.009	0.018 ± 0.012
C16 : 0	Palmitic acid	23.714	0.745 ± 0.100	0.946 ± 0.538	0.814 ± 0.235	0.926 ± 0.184
C17 : 0	Margaric acid	28.721	0.036 ± 0.016	0.044 ± 0.030	0.049 ± 0.036	0.064 ± 0.045
C18 : 0	Stearic acid	35.921	0.583 ± 0.285	0.846 ± 0.611	0.997 ± 0.587	1.125 ± 0.572
C20 : 0	Eicosanoic acid	48.923	0.012 ± 0.001	0.014 ± 0.003	0.015 ± 0	0.019 ± 0
C21 : 0	Heneicosanoic acid	56.060	0.004^a^	ND	ND	ND
C22 : 0	Docosanoic acid	63.220	0.011 ± 0.007	0.013 ± 0.003	0.010 ± 0.007	0.015 ± 0.003
C23 : 0	Tricosanoic acid	70.271	0.003^a^	ND	ND	ND
C24 : 0	Lignoceric acid	77.220	0.009 ± 0.006	0.010 ± 0.001	0.023 ± 0.002	0.043 ± 0.010
C26 : 0	Hexacosanoic acid	82.293	ND	ND	0.015 ± 0.002	0.023 ± 0
Total saturated lipid content			1.831	2.408	2.032	2.352

FA ID: fatty-acid identification; RESV: resveratrol; R.T.: retention time; ND: not determined. ^a^Fatty acid detected in only one independent experiment. MCF-7 and MDA-MB-231 (1 × 10^7^) cells after resveratrol (80 and 200 *μ*M, respectively) or DMSO 0.1% (control) treatment for 24 h were subjected to lipid extraction. Lipid extracts were analyzed by GC/MS. Results are expressed as mean ± S.E.M., *n* = 2.

**Table 2:** Unsaturated lipid profile of MCF-7 and MDA-MB-231 cells after resveratrol treatment.

FA ID	Lipid name	R.T.	MCF-7		MDA-MB-231	
			Control	RESV	Control	RESV
			(*μ*g/*μ*L)		(*μ*g/*μ*L)	
C16 : 1n9	7-Hexadecenoic acid	21.867	0.027 ± 0	0.040 ± 0.025	0.112^a^	0.095^a^
C16 : 1n7	Palmitoleic acid	22.104	0.223 ± 0.133	0.252 ± 0.061	0.093 ± 0.005	0.131 ± 0.030
C17 : 1n7	Cis-10-heptadecanoic acid	27.261	0.027 ± 0.014	0.026 ± 0.01	0.023 ± 0.005	0.038 ± 0.013
C18 : 2n6	Linoleic acid	32.871	0.036 ± 0.012	0.038 ± 0.002	0.038 ± 0.012	0.056 ± 0.001
C18 : 1n9c	Oleic acid	33.820	0.542 ± 0.076	0.831 ± 0.478	0.460 ± 0.014	0.718 ± 0.206
C18 : 1n9t	Elaidic acid	34.094	0.419 ± 0.168	0.334 ± 0.002	0.162 ± 0.005	0.234 ± 0.036
C20 : 4n6	Arachidonic acid	43.802	0.073 ± 0.014	0.102 ± 0.064	0.123 ± 0.071	0.235 ± 0.159
C20 : 5n3	Eicosapentaenoic acid (EPA)	44.181	0.011 ± 0.001	0.019 ± 0.016	0.040 ± 0.020	0.060 ± 0.036
C20 : 3n6	8, 11, 14-Eicosatrienoic acid	45.067	0.016 ± 0.001	0.025 ± 0.020	0.022 ± 0.008	0.053 ± 0.031
C20 : 2n6	11, 14-Eicosadienoic acid	47.228	0.005^a^	0.007 ± 0.004	0.007^a^	0.012 ± 0.003
C20 : 1n9	Cis-11-Eicosaenoic acid	48.739	0.038 ± 0.013	0.042 ± 0.030	0.013^a^	0.011 ± 0.007
C20 : 1		49.254	0.036 ± 0.025	0.027 ± 0.012	ND	0.002^a^
C22 : 6n3	4, 7, 10, 13, 16, 19-Docosahexaenoic acid (DHA)	57.155	0.052 ± 0.010	0.086 ± 0.057	0.084 ± 0.049	0.160 ± 0.108
C22 : 1n9	Erucic acid	61.184	0.014 ± 0.010	0.014 ± 0.004	0.010 ± 0.004	0.008 ± 0.004
C24 : 1n9	Nervonic acid	75.325	0.034 ± 0.019	0.038 ± 0.013	0.012 ± 0.006	0.019 ± 0.002
Total unsaturated lipid content			1.553	1.881	1.199	1.832

FA ID: fatty-acid identification; RESV: resveratrol; R.T.: retention time; ND: Not determined. ^a^Fatty acid detected in only one independent experiment. MCF-7 and MDA-MB-231 (1 × 10^7^) cells after resveratrol (80 and 200 *μ*M, respectively) or DMSO 0.1% (control) treatment for 24 h were subjected to lipid extraction. Lipid extracts were analyzed by GC/MS. Results are expressed as mean ± S.E.M., *n* = 2.

## Data Availability

The data used to support the findings of this study are available within the article.
